# Valorisation of By-Products from Soybean (*Glycine max* (L.) Merr.) Processing

**DOI:** 10.3390/molecules25092129

**Published:** 2020-05-01

**Authors:** Alessandro Colletti, Andrea Attrovio, Luisa Boffa, Stefano Mantegna, Giancarlo Cravotto

**Affiliations:** 1 Department of Drug Science and Technology, University of Turin, Via P. Giuria 9, 10125 Turin, Italy; alessandro.colletti@unito.it (A.C.); andrea.attrovio@edu.unito.it (A.A.); luisa.boffa@unito.it (L.B.); stefano.mantegna@unito.it (S.M.); 2 Centre of Bioanalytical Research and Molecular Design, Sechenov First Moscow State Medical University, 8 Trubetskaya ul, 119991 Moscow, Russia

**Keywords:** soybean curd residue, okara, nutritional composition, functional foods, biovalorisation

## Abstract

In recent years, increased awareness of the health benefits associated with consuming soy-based foods, knowledge of milk-related allergies and a move towards more sustainable food production have led to an increase in the number of available soy-based products. The biggest producers in the world, the USA, South America and China, are from the Pacific region. This enormous production is accompanied by the accumulation of related by-products, in particular, a substance that is known as okara. Okara is a paste that is rich in fibre (50%), protein (25%), fat (10%), vitamins and trace elements. Its proper use would lead to economic advantages and a reduction in the potential for polluting the environment. Its high fibre content and low production costs mean that it could also be used as a dietary supplement to prevent diabetes, obesity and hyperlipidaemia. Chemical or enzymatic treatment, fermentation, extrusion, high pressure and micronisation can all increase the soluble fibre content, and thus improve nutritional quality and processing properties. However, the product also degrades rapidly due to its high moisture content (70–80%), which makes it difficult to handle and expensive to dry by conventional means. The aim of this paper is therefore to thoroughly study the existing literature on this subject in order to develop a general protocol for okara exploitation and valorisation. A cost/benefit analysis could drive the design of eco-friendly, sustainable protocols for the preparation of high-value nutritional products.

## 1. Introduction

### 1.1. Use of By-Products in the Food Sector

The ever-increasing production of food requires a huge amount of resources, and raw materials are not yet fully exploited. This leads to the generation of waste, which adds to the waste generated by the leftovers of consumption [1]. Reducing food waste and making suitable use of resources can help to meet the demand for the estimated 60% higher food production that will needed by the world’s population in 2050. Global food waste also contributes significantly to environmental issues, because of its extensive use of energy and resources, as well as the associated greenhouse gas emissions [2].

There are conspicuous food losses within the food chains of the most developed countries, including 39% during food production and a significant 42% by the consumer [3]. A large number of by-products of the various phases of food production have been studied to find ways to limit food production’s environmental and economic impact and researchers have experimented with new processes for the recovery of valuable components. Moreover, the conversion of many primary metabolites can generate new resources [4,5].

### 1.2. Soybeans: Characteristics, Processing and Use

**Figure 1:**
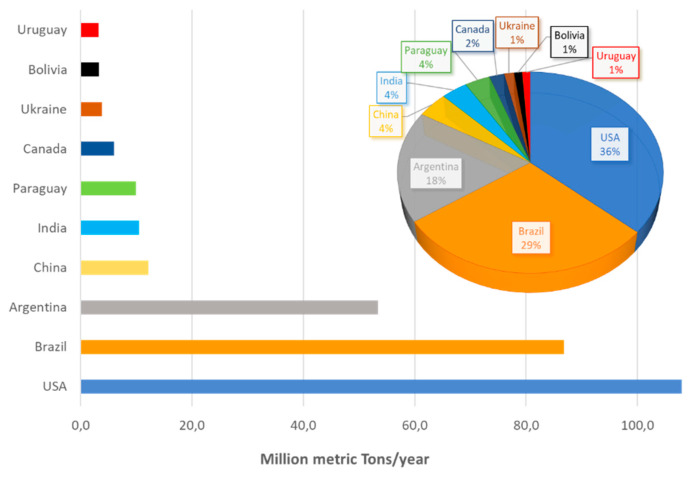
World leaders in soy production, based on annual data released by FAOSTAT.

Soybeans (SBs) are currently one of the most important food crops worldwide. Although they originated in Asia and have been cultivated there for thousands of years, 7 of the top 10 producers are presently found on the other side of the Pacific region, in North and South America [6] (Figure 1).

Despite the ancient tradition of soy foods, it is only in the last 15 years that they have also been shown to be beneficial in the prevention and treatment of various chronic diseases [7]. However, individuals can develop an allergy to this important legume [8].

It is now well established that SBs are an abundant source of proteins due to their high nutritional value and excellent physical and chemical properties (Table 1). Moreover, SBs and SB products are rich sources of minor non-nutrient components with potential health benefits, which are often referred to in the literature as phytochemicals. Within this group of substances, we find biologically active proteins and peptides, such as protease inhibitors (which include trypsin inhibitors, low molecular weight proteins that bind trypsin and thus interfere with protein hydrolysis during digestion) [9], lectins (tetrameric glycoproteins that make up 1–2% of the seed and that are able to agglutinate red blood cells and, once ingested, stimulate the secretion of pancreatic enzymes and enterocytes, interfering with the intestinal absorption of nutrients) and lunasin (a recently discovered peptide, a major component of Bowman-Birk’s protease inhibitor, particularly effective in suppressing carcinogenesis) [10]. SB products are also important sources of isoflavones, phytosterols, phytic acid and saponins. In traditional nutritional theory, many of these components have been considered anti-nutrients. However, in the last two decades, it has been discovered that they can exert beneficial health and therapeutic effects, from cholesterol-lowering effects to anticancer effects, a controlling effect of diabetes mellitus and the reduction of postmenopausal osteoporosis [11].

As far as processing is concerned, SBs are first classified, cleaned, dried at about 10% humidity and split to remove the shell. SB shells are processed to create fibre additives for bread, cereals, snacks and livestock feed. After husking, the beans are rolled into fat-rich flakes that can be used in animal feed or processed into wholemeal flour for food use. Crushing breaks up the oil cells of the seed, improving the oil-extraction process. The next step is to extract the raw oil, which is then refined to produce cooking oil, margarine and pastry fat. 

The defatted soy flakes are used to produce animal feed and form the basis of a variety of products for human consumption, including SB meal, SB concentrates and SB isolates. These products are widely used in food to help retain moisture and improve shelf life, as well as acting as emulsifiers and meat substitutes in food products. SB meal is produced by grinding the defatted flakes. The protein content of the flour is about 50%, which makes it a source of protein, while also improving the colour of the crust and the shelf-life of baked goods.

SB isolates are produced in a chemical process that removes most proteins from the defatted flake, resulting in a product with about 90% protein content that is free from fibre and carbohydrates. Isolates are used in many dairy-like products, including cheese, milk, non-dairy frozen desserts, coffee whiteners and meat products. SB concentrates, on the other hand, are prepared by removing soluble sugars from defatted flakes and contain about 70% protein and retain most of the dietary fibres of the seed. They are used in protein drinks and as a basis for soups and sauces. SB meal and soy-protein concentrates are used in meat products, mainly because of their fat- and water-absorbing properties [13]. Nutritional values of traditional fermented soy-based foods are reported in Table 2.

SB-based foods are generally divided into two categories: unfermented and fermented foods. Traditional unfermented soy-based foods include fresh SBs, whole dried SBs, soy nuts, SB sprouts, whole SB meal, soymilk and soymilk products, such as tofu, okara and yuba. Fermented products include tempeh, miso, soy sauces, natto and finally tofu and fermented soymilk products [15].

### 1.3. Okara: between Production and Consumption

**Figure 2:**
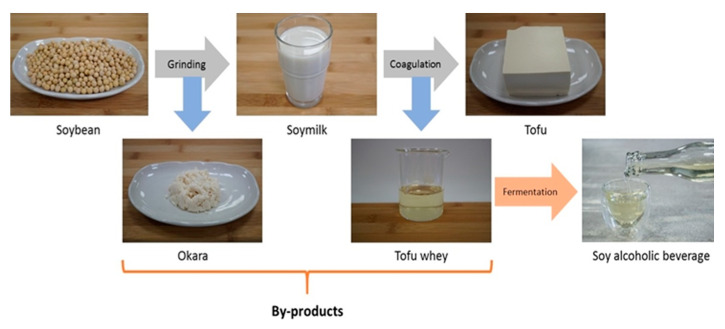
Soybean by-product production.

Increased awareness of the health benefits associated with SB food consumption, knowledge of milk-related allergies and a move towards more sustainable food production have led to an increase in the amount of soy products available. Soymilk is one of those products, the production and consumption of which has increased in most countries and is continuously accompanied by the accumulation of the okara by-product. Current statistics on soymilk production are not widely available. In 1983, soymilk production was estimated at about 1 million tonnes [16], but it has dramatically increased in recent decades. In 2006, SB beverage production reached over 1 million tonnes in Western Europe, North America and Japan alone [17]. Traditional soymilk is produced at a SB to water ratio of 1:5, although sweetened and flavoured soy drinks are made at a seeds-water ratio of up to 1:20. However, it can be estimated that about 170,000 tonnes of okara are produced from 1 million tonnes of soymilk for dairy-type soymilk (protein content 3.5%) at a seed-to-water ratio of 1:7, and assuming that 1.2 tonnes of SB are produced per tonne of SB during soymilk production [18]. Tofu and other soy products are not included in this estimate, but they are similarly based on primary soymilk production (Figure 2). It is therefore reasonable to conclude that several million tonnes of okara are produced each year [19,20,21].

## 2. Composition of Okara, A By-Product of Soymilk Production

Soybean curd residue (SCR), also known as okara (Japan), biji (Korean) or douzha (Chinese), is a by-product of SB processing. For each kilogram of SB used to produce soymilk or tofu, about 1.1–1.2 kg of okara are formed [22]. Its main components are broken cotyledon cells and the coating of SBs. Currently, SCR is treated as waste and is either used as feed, fertiliser or is landfilled. In Japan in particular, most of this is burned, creating carbon dioxide [23].

**Figure 3:**
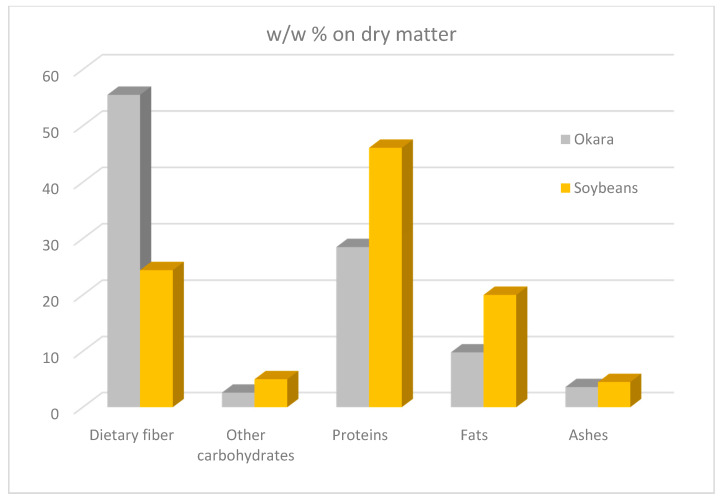
Dry composition of okara, compared to SBs.

As indicated in Table 1, okara consists of about 80% water, 3.5 to 4.0% protein and most of the insoluble SB components. Its high moisture content means that okara also contains water-soluble components. Its exact composition mainly depends on the variety of SB used, the incidence of sunshine and other environmental factors, as well as the processing conditions used during soymilk production. Therefore, the characteristics of the water-soluble fraction may vary depending on the raw material used [24]. When moisture free, okara contains near 10% fats, 55% crude fibre and 30% protein (Figure 3) [25].

### 2.1. Dietary Fibre

Although okara has a high moisture content (nearly 70–80%), most of the water is linked to the dietary fibre, resulting in a pasty texture that is similar to waterlogged sawdust. Fibre, mainly insoluble fibre (in the form of cellulose and hemicellulose), accounts for most of the dry matter content (40–60%) [25] which can be fermented by microbes in the large intestine, although it cannot be digested in the small intestine. By comparison, the amount of free carbohydrates (such as arabinose, glucose, galactose, fructose, sucrose, raffinose and stachyose) is low (4–5%) and the lack of fermentable carbohydrates is the main factor limiting efficient fermentable microbial growth. In particular, it contains 1.4% stachyose and raffinose, which can cause flatulence and swelling in some individuals. The monomers that make up the polysaccharide fraction of the cell wall are mainly galacturonic acid, galactose, arabinose, glucose, xylose, fucose and a small amount of rhamnose and mannose [26] (Table 3). Their influence on the water retention and swelling capacity of okara places it is a potential source of texturizing additives [27]. Mateos-Aparicio et al. have subjected okara to high hydrostatic pressure (HHP) leading to the amount of soluble dietary fibre increasing more than 8-fold, which is useful in ensuring that okara has anti-inflammatory and anti-carcinogenic effects on the digestive tract [28].

A combination of okara and soft wheat flour has been found to result in an increase in the contents of protein, dietary fibre and isoflavones, compared to the use of soft wheat alone [29]. The importance of dietary fibre is linked to the regulation of intestinal functions [30]. Their presence in okara has been associated with potential hypolipemic and hypocholesterolaemic effects [19,31], as well as hypocaloric effects, thus making it effective and useful for the improvement of metabolic syndrome [32]. The prebiotic action that it provides is also interesting [33]. Finally, research on Syrian hamsters fed with okara has suggested how its main components, dietary fibres and proteins, may be related to a reduction in total lipids and cholesterol and an increase in faecal production in animals fed a fat-rich diet [34].

### 2.2. Protein Component

Proteins make up between 15.2 and 33.4% of okara (dry matter). The main proteins are globulin 7S and globulin 11S [35]. The okara protein isolates contain all of the essential amino acids, and have a protein efficiency index that is even higher than that of soymilk (2.71 vs. 2.11) and tofu, but with low water solubility [36,37] (Table 4). It has also been found that the protein fraction of SCR is able to withstand complete digestion by the gastrointestinal enzymes, pepsin and pancreatin (the latter of which mainly consists of trypsin, amylopsin and steapsin). The low molecular weight fraction (less than 1 kDa) of these digestible peptides has a more potent ability to inhibit the angiotensin-converting enzyme and shows great antioxidant activity, probably because of its high percentage of hydrophobic amino acids [38]. Trypsin inhibitors can range from 5.2 to 14.4% of the protein content, although they can be inactivated with appropriate heat treatment [39].

The bioconversion of high molecular weight okara proteins into smaller proteins may increase the solubility of protein isolates and generate bioactive peptides or amino acids. Chan and Ma [40] have shown that the solubility of the protein fraction, when treated with acid, can increase considerably (recovery of 53% when extracted at pH 9 and 80 °C for 30 min), and thus lead to improvements in other functional characteristics, such as emulsifying and foaming properties. In addition, it has been found that maximum protein recovery (93.4%) is achieved with an okara powder fineness of less than 75 µm [41].

Trypsin inhibitors can also be degraded by microorganisms in order to improve the nutritional quality of the residue. However, microorganisms can catabolise proteins and amino acids, reducing the amount of essential amino acids present. The various effects of fermentation on the molecular weights of peptides, the amino acid profile and the inhibitory activity of trypsin should therefore be considered, as they can influence the overall functional characteristics (such as solubility and foaming properties) and bioactivity of fermented okara.

### 2.3. Lipid Fraction

SCR also contains a considerable amount of lipids, between 8.3 and 10.9% (dry matter). Most fatty acids are mono- or polyunsaturated and consist of linoleic acid (54.1% of total fatty acids), oleic acid (20.4%), palmitic acid (12.3%), linolenic acid (8.8%) and stearic acid (4.7%) [27].

Lipoxygenase and hydroperoxide lyase react with unsaturated fatty acids, mainly linoleic acid, during SB grinding, leading to the formation of aromatic compounds, such as hexyl and nonyl aldehydes and alcohols. These low-detection-threshold odours represent the aromas of raw soymilk. Since these enzymes are normally denatured at temperatures above 80 °C, the Chinese method of making soymilk (in which the SBs are ground before the filtrate is boiled) produces an okara in a greener manner [42]. The variant obtained using the Japanese procedure for soymilk production is therefore relatively more palatable and probably has lower trypsin-inhibitor content, meaning that it can be more easily used for cooking and processing [39]. This may explain the phenomenon by which okara is often sold as a packaged product in Japanese supermarkets, but rarely found in Chinese shops.

Fermentative microorganisms can metabolise fatty acids and their derivatives to produce more desirable aromatic compounds. Quitain et al. have investigated the recovery of okara oil components via extraction with supercritical carbon dioxide, modified with ethanol [43]. The results indicated that 63.5% oil-component recovery was obtained at a relatively low temperature of 40 °C and a pressure of 20 MPa in the presence of 10% mol EtOH. This oil component mainly consisted of fatty acids, phytosterols and traces of decadienals. EtOH proved itself to be useful in increasing the yield and amounts of phenolic compounds and the two primary soy isoflavones, genistein and daidzein, in the extracts. These compounds are known antioxidants that can increase both the stability and value of the oil, making the process attractive for the cosmetic, pharmaceutical and food industries.

### 2.4. Isoflavones

Isoflavones are present in many legumes, and SB can be considered an important source [18,44]. Wang and Murphy have shown that there are more aglycons in okara than in soymilk. Generally, the isoflavone content in SCR varies between 12% and 40% of the original isoflavone content in the beans. This leads to isoflavone concentrations in the pulp of between 0.02% to 0.12% (dry weight), depending on SB type [29]. The role of isoflavones as phytoestrogens has made them a topic that has been widely discussed in the literature, as they are attributed with important activity against hormone-derived cancers, osteoporosis and menopausal syndrome disorders [45,46,47].

SBs contain up to twelve different categories of isoflavones, classified into three main groups (daidzein genistein and glycitein), all of which can take four different forms: aglycones (15.4%), β-glucosides, malonyl-glucosides (28.9%) and acetyl-glucosides. Okara may contain the same twelve isoflavones, although the processing conditions during the production of soymilk and its residue may change the original isoflavonic profile [48]. Isoflavones are polyphenols with a structure that is similar to flavones [49,50]. Both flavones and isoflavones are subclasses of flavonoids, one of the largest groups of polyphenols [51,52].

The total concentrations of isoflavone and the different forms of isoflavone in SBs and their products depend on SB variety, its cultivation, the processing and storage conditions [44,53,54,55] (Table 5). Malonyl-glucosides and β-glucosides are the primary forms in SBs. However, these components can be transformed into aglycones and acetyl glucosides during processing, following either enzymatic conversion or thermal stress [56]. The most common chemical changes in isoflavones include the decarboxylation of malonyl to acetyl-glucosides, and the ester hydrolysis of either acetyl or malonyl-glucosides into β-glucosides. In addition, the splitting of their glucosidic bond leads to an increase in the amount of aglycones [57], which show greater bioavailability in humans [58].

Fermentative microorganisms are also able to secrete β-glucosidase, thus bioconverting isoflavonic glucosides into aglycones, and are thus an opportunity for value addition [59].

Producers use different methods for the small-scale and large-scale production of soymilk and okara. This change in processing conditions may affect the isoflavonic profile in the resulting products. The most significant differences are in the temperatures used during SB immersion, the, either hot or cold, grinding of the SB suspension, cooking temperature and sterilisation before or after separating soymilk from its residue [60,61,62]. Another factor that complicates isoflavone behaviour in okara is their interactions with other matrix components, such as non-covalent interactions between polyphenols and macronutrients, mainly proteins [63,64].

The health effects of polyphenols, including a reduction in the risk of cardiovascular and cancer diseases [47], antioxidant and anti-inflammatory properties [65,66] and useful effects against type 1 and type 2 diabetes mellitus [67], have been widely proven.

The exploitation of by-products for the recovery of polyphenols has aroused particular interest with a view to contributing to more sustainable agriculture and food production [68]. In fact, by-products are often very rich in phenolic compounds, due to their presence in peels and seeds, which are often retained in the residues. Their relatively low water solubility and tendency to associate with other components may contribute to these by-products being rich in polyphenolic content. The potential applications of polyphenols are plentiful: food flavourings and colours, bioactive ingredients for health and antioxidant stabilisers.

Several conventional and unconventional technologies have been proposed for the separation of these high-value components. Conventional solid-liquid extraction commonly uses hydroalcoholic mixtures [69]. However, many other solvents, such as acetone, acetonitrile, methanol and ethyl acetate, are still widely studied in the extraction of polyphenols, due to the relatively easy solubilisation offered by these solvents and mixtures [70]. Acid, alkaline and sub- or supercritical fluid extraction are common alternatives. Modern technologies, such as pulsed electric fields, ultrasound-assisted extraction and microwave-assisted extraction, have been suggested as means to increase yields and overcome some difficulties in polyphenol extraction. Examples of possible obstructions include kinetic limitations in cell matrix extractions, component instability and solvent residues in the final product.

Soy molasses, a by-product of soy-protein concentrate preparation, is a popular starting material for the production of isoflavone. Being an alcoholic extract of soy flakes, it contains isoflavones in a slightly more concentrated form. However, many patented processes use SBs or SB meal as a starting material when the recovery of isoflavones from side products, such as okara, would require less valuable resources.

### 2.5. Nutritional and Anti-Nutritional Elements

Soyasaponins are a group of non-volatile amphiphilic molecules that are present in a wide variety of legume seeds, such as SBs, peas, lentils and lupins [71]. Soy-based products are the main food sources of soyasaponins [72]. These are mainly contained in the cells of SB cotyledons and released into the okara after processing. It was reported by Gurfinkel and Rao, in 2003, that they possess immunostimulating, antiviral, hepatoprotective and chemopreventive properties [73].

Other components include minerals, lignans, coumestans (the latter two belonging to the category of phytoestrogens, which are inhibitors of enzymes involved in the metabolism and biosynthesis of estrogens, such as aromatase, 17-β-hydroxysteroid reductase, sulfatase and sulfotransferase), phytosterols and phytates (responsible for the chelation of calcium and iron in the intestine).

Anti-nutritional factors present in okara, such as phytates, saponins and trypsin inhibitors, limit its use in animal feed [74]. Fermentative microorganisms can metabolise these and other factors, such as allergenic proteins (containing glycine and β-conglycine), by providing a final fermented product of improved nutrition and digestibility [75].

Okara also contains a variety of minerals, such as potassium, calcium and iron [27,76], whose consumption can improve human health (see Table 1). For example, potassium reduces systolic blood pressure [77], copper promotes antioxidant defence and immune function [78], and magnesium mitigates hypertension problems and helps prevent diabetes complications. The absence of iron has been associated with fatigue and an increased risk of cardiovascular/thromboembolic events [79], while calcium prevents osteoporosis and fractures in adulthood and old age [80].

## 3. Production and Use of Okara

Soymilk can be made from whole SB or fat soy flour. Its production usually consists of five main stages:

(1) SB washing to remove impurities
(2) SB soaking/hydration for 12 h at 25 °C, then draining and rinsing with water
(3) cooking at 98 °C for 5 min, with the aim of both sterilising and improving aroma and nutritional value via the inactivation of trypsin inhibitors
(4) grinding in a blender with distilled water (1:10 ratio *w*/*v* SBs/water) for the preparation of a slurry
(5) separation of the slurry into soymilk (water-soluble SB extract) and okara by mechanical means (usually filtration) [16,21,81].

The moisture content primarily depends on the soymilk-okara separation method. Wet okara, due to its high moisture content, deteriorates very quickly, making its use difficult. Every element in soymilk processing is fundamental. In the Japanese manufacturing method, whole soaked SBs are first cooked and then ground and filtered. In the Chinese method, raw SBs are first ground and then extracted with water, filtered and finally heated [82].

Two main approaches to the use of okara in foodstuffs have been described in the literature. In the first, whole SCR is used as a component in finished products. In the second approach, several constituents are isolated from it and then used as ingredients.

The inclusion of okara as a complete ingredient in foodstuffs often requires the drying of the fresh residue. Okara is a difficult material to dry because of its high tendency to agglomerate, leading to spattering phenomena, and the low energy efficiency of the drying process [83,84,85]. In the dry state, it is used to improve the consistency of a product, since it has a good retention capacity for water and oil, and to enrich foods with nutritional components, such as fibre and proteins. Examples of products that contain whole SCR are: a soy-based snack [86], a cheese bread [87] and a French bread [88].

The addition of wet okara to food products is being considered even less than previously by producers. Its direct incorporation into feed or food is possible, but it is limited by the presence of enzyme inhibitors, regardless of whether SBs have been heat treated before grinding [28,74,89]. Turhan et al. and Su et al. have described its use in the production of low-fat beef burgers [90,91] and to reduce the fat content in a coconut-based baked snack [92]. Furthermore, in 2000 Rinaldi et al. have introduced freshwater products that are enriched with okara [29].

As regards the use of SCR ingredients, more attention has been paid to the polysaccharide fraction. Its polysaccharides have been characterised in detail by several authors, as described in Section 2.1. Okara fibres, which are mainly insoluble, have a high water- and oil-holding capacity, and also swell in water. The indigestible fraction shows good suitability for fermentation by *bifidobacteria*, indicating that it may potentially be used as a prebiotic ingredient [93]. To increase fibre solubility (from 38.1% to 64.8%), okara is either treated with enzymes and a high-pressure process (HPP) [28] or by extrusion [19,94].

Regarding the protein fraction of okara, past research has focused on methods of extraction and modification that can make it suitable as an ingredient [36,37,40], while recent literature has reported detailed characterisation and composition [39,95]. In addition, Vishwanathan et al. have investigated the extractability of proteins in SCR and the production of a protein concentrate by membrane separation [96]. A study on peptide preparation via protease hydrolysis has reported that the obtained products showed antioxidant activity [97].

Finally, okara has been studied for its potential ability to provide natural antioxidants. Methanol, acetone and water have been used to extract polyphenols and oligosaccharides, which are precious components with antioxidant and prebiotic properties [45,98,99,100].

## 4. Application of Okara in Functional Foods

The chemical and nutritional qualities of SB, which are, in particular, high protein content, a fatty composition that is rich in unsaturated fatty acids (for example, linoleic acid), and high isoflavones contents, have, over the years, aroused great interest from the food industry. Specifically, the incorporation of soy-derived ingredients into a variety of products with the aim of providing beneficial properties to the body has been the focus. These are called ‘functional foods’ [101]. In Western countries, for example, water-soluble SB extract (soy drink or soymilk), one of the main products, has been used as an important alternative for people who are intolerant to lactose or allergic to cow’s milk proteins [102]. Furthermore, other traditional soy-based foods, such as tofu, miso, tempeh and soy sauces, should be mentioned in the same context.

### 4.1. Production of Food for Human Consumption

Even okara has been used for many years for food purposes in China and Japan, both in its raw and processed forms, to more easily provide a fair intake of fibre and protein. SCR has solvent-binding properties, making it an ideal low-cost ingredient with which to increase yields in meat products [24]. It has also been shown to have a positive effect on the shelf life of chocolate cookies at an optimal concentration of 5%, as well as preventing syneresis in cheese ravioli filling during freezing and defrosting. At the same time, its insipid taste allows it to be used at relatively high levels without adversely affecting the taste or texture profiles of meat and bakery products.

Okara can partially replace wheat flour, SB flour and other ingredients in food production to increase fibre and protein contents. Several studies have indicated its usefulness in the production of bread, pancakes, puff pastries, pasta, candies, drinks, sausages and nutritional flour.

In a study by Wickramarathna et al., bread that was made by replacing wheat flour with 10% okara powder had almost the same sensory qualities and physical-chemical characteristics as normal bread [103]. A significant difference in crust colour was found in the control (white) and the 10% SCR-enriched bread (red), which had a caloric value that was higher (15.9 kJ/g) than the control value (14.4 kJ/g), due to the higher protein and fat content in the okara.

Suda et al. have produced pancakes and bread with the addition of okara powder (50.4% dietary fibre, 21.3% vegetable protein and 0.45% calcium) to the food ingredients, with the aim of developing fortified foods for medical use [104]. Three different types of bread were processed, including the 10% SCR bread, and other additives and preservatives were needed to promote yeast fermentation and allow storage at room temperature. After freezing without preservatives, the flavours of the three breads were modified. A soft pancake with 20% okara was prepared using a mixture of pancake powders: the preservative-free pancake was suitable for storage in the refrigerator before being eaten, while both the fresh pancakes and those containing the preservatives made with 20% okara were acceptable as supplements for the hospitalised elderly. The study showed that the soft pancake that was based on SB pulp and contained 40% water was more useful as a convenient supplement for dietary fibre, vegetable protein and calcium than the okara breads.

The quality of bread produced with the addition of okara is significantly increased by adding enzymes (pentosanase, lipase and glucose oxidase). The increase in powder substitution from 4% to 8% has also been found to lead to a quality increase [105]. Bread with 5% fresh SB pulp fibre, treated with 1% NaOH for 1 h and 1% HCl at 60 °C for 2 h, had a quality and appearance that was very similar to those of normal bread [106]. Okara can also replace part of the wheat flour needed to make cookies and cakes [107,108,109,110].

Blown food is produced by subjecting cereals, potatoes or SBs to preheating at 300–400 °C in an autoclave at high pressure with steam (15 bar). Using okara and starch as raw materials, blown soy-food can be processed via extrusion and frying. The fresh residue was first immersed in water at pH 3–5 and 80–100 °C for 2 h. Subsequently, after neutralisation, filtration, drying and crushing, the okara powder was mixed with starch, water, 2% salt and 1% spices. The optimal conditions for the extrusion were: a temperature of 160 °C, and the presence of 30% water and 40% starch. The extruded food was fried at 180 °C for 40 s. The processed product had a crisp texture and an attractive taste, and was devoid of the typical okara taste, which is reminiscent of broad beans [111]. 54% starch, 8% SCR mixed with sweet potato residue (6:4 ratio), 31% water and 7% additional materials were used to give a high-quality product via frying at 160–165 °C for 3.5 min after extrusion [112]. Finally Yu has proposed the use of 100 g okara, 500 g potatoes, 150 g wheat flour and 20 g powdered milk as raw materials, which, after fermentation with yeast at 28 °C for 1 h, drying at 60 °C for 1–2 h and frying at 180 °C for 1 min, afforded an okara-fibre-based food [113].

Dried noodles that contain dietary fibre and have a lower glycaemic index can also be prepared using SB pulp [114]. The noodles were of good cooking quality when okara was added at 9% (with a particle size of 100 mesh), 0.25% sodium alginate and 4% salt [115]. Bedani et al. have increased the nutritional and functional properties of soy yoghurt with the addition of okara [116], while Waliszewski et al. have added it to corn tortillas to improve their amino acid profile [117]. In Argentina, SCR-containing candy (nougat) has been tested as well as peanuts, glucose, hydrogenated oil, sugar and natural essences with the aim of increasing the supply of available vegetal proteins [118]. In addition, it may also be possible to produce steamed bread [119], a healthy drink [120], sausage [121] and sliced vegetables [122].

On the other hand, the development of a composite okara powder is an ideal way to use SCR. Xie and Li have invented an enriched nutritional flour that is suitable for diabetics; fresh okara was dried and ground into a powder (80–200 mesh), then mixed (5–25%) with either wheat flour, wheat gluten or buckwheat flour to improve food-processing properties [123]. This composite flour can partially replace wheat flour in the daily preparation of foods, such as bread, steamed bread and pasta for diabetics. Rotem and Almog have developed a premixed powder that contained protein-rich SCR for health-food applications; ground and dried okara (70 mesh) was mixed with gluten, in proportions of between 3:1 and 12:1 [124]. The product contained soy proteins, at between 10% and 30%, and total proteins were between 15% and 50%.

### 4.2. A Study on Paste Production

**Figure 4:**
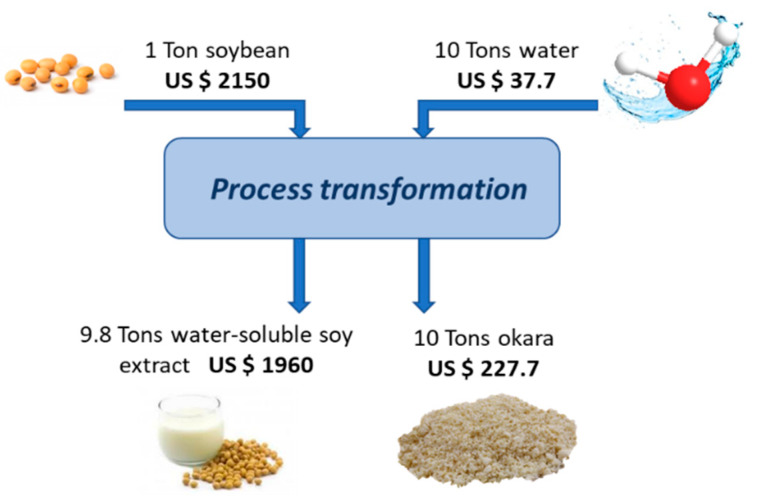
Costs (US $/Ton) of the production processes of water-soluble SB extract (soymilk) and okara (data from Guimarães et al.) [126].

Even SCR, once dried, is an excellent alternative for incorporation into food products such as pâté, process adjuvant or food fortification [125]. An economic analysis of the potential exploitation of okara for food enrichment has been carried out. 1 kg of SB and 10 L of water are used in the production process of soymilk, resulting in 1.2 kg of okara (Figure 4). Therefore, the okara generated by the process corresponds to 10.9% of the raw material used and is usually discarded without generating financial resources. An estimation of the total cost of processing SBs to produce soymilk means that 227.7 US $ per tonne of raw material is discarded during the generation of okara. The feasibility of incorporating okara into food is therefore a solution for waste, whose disposal generates costs and environmental impact.

Vegetable paste is a homogeneous mixture of vegetables to which various aromas are added to improve sensory properties. Once packaged and subjected to a suitable thermal process (4 °C/48 h), it is eaten cold. Guimarães et al. have prepared and analysed 3 formulations with different SCR contents (F1 at 33.87, F2 at 43.45, F3 at 50.60 *w*/*w* percentage, respectively), in which carrots and mayonnaise were liquefied to produce a homogeneous mass and to which, subsequently, SB pulp, spices and lemon juice were added in appropriate quantities to give a product with good acceptability and nutritional quality. The increase in the okara fraction in the formulations led to an increase in the total dietary fibre, protein and lipid contents, as well as the energy of the samples (Table 6). The calcium, potassium and total isoflavone contents also increased with the incorporation of greater amount of okara in the formulations (from F1 to F3).

The formulation of the vegetable paste enriched with 33.9% okara (F1) has a low energy value (89.65 kcal/100 g) and lipid percentage (5.6%), but high protein (3.1%), β-carotene (0.411 mg/100 mL), dietary fibre (5.8%) and isoflavone (0.15 μmol/g fresh matter) contents, and demonstrated high sensory acceptability. In conclusion, the use of okara in the development of a vegetable paste is an interesting and feasible alternative means to increase the nutritional value of a product and to effectively exploit the residue generated by the production of soymilk.

### 4.3. Fibre Recovery and Enhancement

The dietary fibre market is highly competitive because of its beneficial effects in reducing the risk of cancer and coronary heart disease [127]. Due to its high fibre content, low costs and the abundant volumes produced, SCR is an excellent raw material from which to recover dietary fibre.

A great deal of research has been published on the supply of okara fibre. In one of these studies, dietary fibre was prepared from wet okara via the following steps: drying, porphyrisation, soaking with alkaline solution, enzymatic hydrolysis, bleaching, precipitation with ethanol and drying. The conditions for alkaline soaking were 4% NaOH at 80 °C for 80 min. The enzymatic hydrolysis of trypsin was performed for 30 min to remove the protein content. Whitening was performed with 3% H_2_O_2_ at 50 °C for 60 min. After processing, the okara fibre was composed of 41% insoluble fibre, 15% soluble fibre, 6% lipids, 1% protein, 2% ash and 8% moisture. The water-retention capacity and hydration capacity were 10.08 g H_2_O/g of product and 18.66 mL H_2_O/g of product, respectively [66]. Li et al. have reported that optimal bleaching was performed with 3 g of H_2_O_2_/100 g of dry okara at pH 10 and 80 °C for 3.5 h [128]. After bleaching, the whiteness of the fibre residue reached 88%, the water-retention capacity increased 1.7-fold and the expansive capacity improved 1.9-fold. The following treatments have been tested to improve soluble fibre yields from okara: extrusion [129,130], high pressure [28], chemical and enzymatic [131,132,133] and fermentation by microorganisms [134].

### 4.4. Applications in Animal Nutrition

SCR contains a high content of proteins and non-fibrous carbohydrates, making it attractive for use with dairy cattle and goats [135]. It is also much cheaper than SB meal and can therefore be used as feed for cattle, pigs, sheep, fish [136] and poultry [137] with the aim of replacing part of their normal feed.

When half of SB meal was replaced with okara to feed dairy cattle and yellow cattle for 30 days, there were no significant differences in milk production, milk-fat content, feed consumption and daily profit (for yellow cattle) between the groups fed on milk and those fed on SB meal. The feeding cost of the okara-fed group was significantly lower than that of the SB-fed group [138,139].

Meeting the high protein requirements of young pigs is a challenge for organic pig producers: the price of organic feed is up to 4 times higher, and has limited availability. Okara is a potential source of alternative organic proteins and its intake in up to 25% of young pigs’ diets had no effect on average daily profit, average daily food intake and gain/feed ratio compared to the control [140].

Many food by-products have a high moisture content and, in order to avoid the high energy costs of drying, are therefore often stored via silage, a forage storage technique that consists of acidifying the plant mass using anaerobic microorganisms, with the aim of preventing other microorganisms from colonising it resulting in the loss of its nutritional value. Therefore, a typical practice in Japan is to mix wet by-products with dry feed to prepare low moisture total mixed ration (TMR) silage. The combination of okara and peanut shells can therefore have a synergistic effect on the silage mix to achieve the right dry matter and fermentable carbohydrates for optimal silage fermentation. Silage with an okara/peanut waste ratio of 78:22 has been found to reduce fibre content and lignification, and improve the efficiency of both silage fermentation and *in-vitro* ruminal fermentation models after 8 weeks [135].

SCR can also be used to produce microbial proteins, which are synthesised by solid state fermentation. During fermentation, the mould degrades the okara fibre into low molecular weight carbohydrates, which are further used by yeasts to synthesise proteins. In addition, some anti-nutritional factors in okara (such as trypsin inhibitors, saponin and lectin) can be decomposed or reduced via fermentation [141]. After solid fermentation for 3 days by a mixed bacterial culture using *Aspergillus oryzae*, *Aspergillus niger* and *Saccharomyces cerevisiae* yeasts, the crude protein content of okara increased by 43.1% compared to the original [142]. It has also been found that crude protein content doubled, compared to the original material, when using okara and wheat bran (ratio 8:2) as a substrate, and *Aspergillus niger*, *Trichoderma viride*, *Saccharomyces cerevisiae* and *Candida utilis* (ratio 1:1:1:3) as a mixed crop, after fermentation at 32 °C for 3 days [143].

### 4.5. Application in Ecological Materials

It has recently been discovered that sedimentation materials, such as okara, wheat bran, rapeseed and flaxseed, can effectively absorb organochlorine compounds. When these were applied to wastewater (pH 10) that contained 0.1 g/L of dichloromethane, this organic solvent was removed from the wastewater with an efficiency of 70–90% after 90 min. High correlation was found between the removal efficiency and the number of spherosomes, which are intracellular particles used to absorb organochlorine compounds [144]. It has also been observed that okara (10 g/L) can absorb 96% Cd^2+^ and 89% Zn^2+^ from water that contained either 50 mg/L Cd^2+^ (pH 6) or Zn^2+^ (pH 7). The maximum absorption quantity was 19.61 mg of Cd^2+^ and 11.11 mg of Zn^2+^ per g of okara, respectively [145]. The use of okara as an adsorbent is therefore an efficient and economical method for the removal of organochlorine compounds and heavy metal ions from wastewater.

Okara is a good material for the production of edible packaging and biodegradable materials. Zhang et al. have separated the fibre from okara using protease and lipase treatments [146]. Edible wrapping paper was then produced by mixing the fibrous material with Chinese sweet potato, dextrin, sucrose and carrageenan, according to the conventional paper-processing method. The stress resistance, flammability and dissolution rates of edible paper were 11.6 × 10^5^ Pa, 138 g/cm^2^ and 7.97 mg/s, respectively. Edible paper had a similar softness to conventional paper, but higher strength and brittleness. Moreover, its water absorption and solubility were higher than that of normal wrapping paper.

In another study, Wen and Liu have prepared an edible film by mixing okara fibre with 2% dextrin and 1.5% glycerol, throwing the mixture on a glass plate and drying it to remove water [147]. Li et al., however, etherified the residue with epichlorohydrin and prepared degradable composites using modified okara and polyvinyl alcohol, with the addition of CaCO_3_ [148]. The water-absorption capacity of composite materials decreased as the degree of etherification increased. Composite materials degraded in soil, with a weight-loss rate of more than 70% after 60 days. Chen has prepared a biodegradable plastic using corn gluten flour and okara. The corn gluten flour was first mixed with okara and the mixtures were extruded into pellets using a twin-screw extruder. The pellets were then extruded again as a final preparation [149]. When 20% okara was mixed in, the sample showed the maximum tensile strength and maintained thermal stability of less than 240 °C. The plastic can be used in agriculture thanks to its low cost and biodegradability.

Finally, okara has a potential application in the ecological preservation of wood, and can be useful as a replacement for copper azole (CuAz) and quaternary ammonium copper salt (ACQ), which are widely used as replacements for copper arsenate chromate (CCA). Enzymatic-hydrolysed okara wood preservatives showed good stability against hot-water leaching.

**Figure 5:**
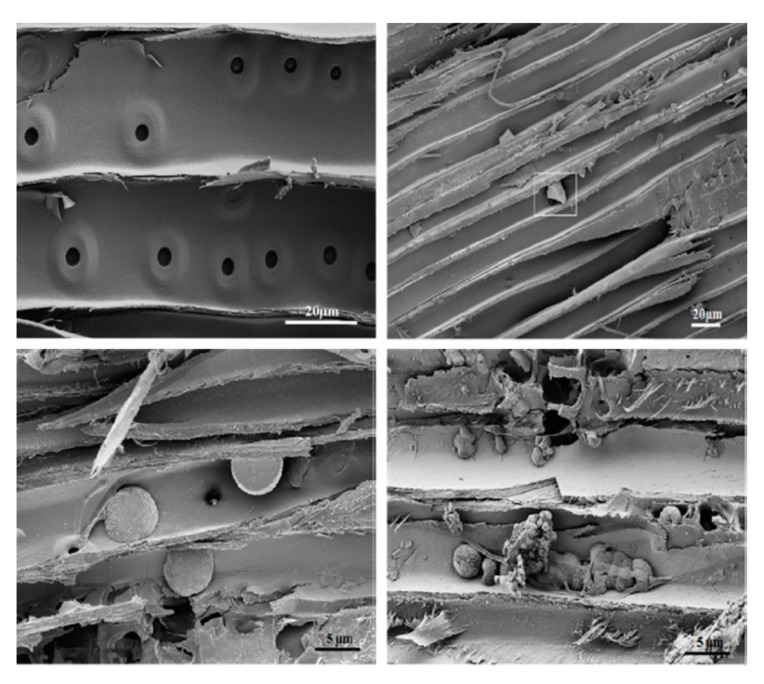
Scanning electron microscopic (SEM) images of control wood block (top-left), wood blocks treated with CC formulations (top-right), leached wood blocks treated with OK/CC (bottom-left) and OK/CC/B (bottom-right) formulations with permission from Elsevier [20].

The leached wood blocks treated with formulations of okara/CuCl_2_ (OK/CC, Figure 5 bottom-left) and okara/CuCl_2_/Na_2_B_4_O_7_ (in the presence of NH_4_OH, used as a dissociating agent) (OK/CC/B, Figure 5 bottom-right) were highly resistant to fungal decay, against both *Postia placenta* and *Gloeophyllum trabeum*, compared to the control and CuCl_2_-treated wood blocks (CC, Figure 5 top-right) especially when okara was hydrolysed by cellulase (Celluclast^®^ with an enzyme loading of 0.1 mL/g) [20].

## 5. Biovalorisation through Fermentation

**Figure 6:**
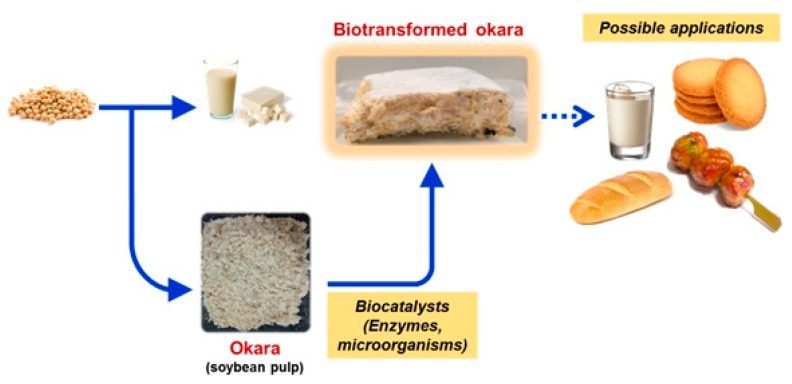
Flowchart illustrating process flow from SB to biotransformed okara, with potential applications in food products (Image credit: Vong Weng Chan) [151].

Okara is rich in carbohydrates, proteins and other nutrients, making it a potentially useful substrate for microbial fermentation. Fermentation can reduce the content of raw fibre, increase the content of soluble fibre, proteins, amino acids and isoflavones, as well as decomposing phytic acid (which is a deposit of phosphorus in the seeds that is indigestible to humans and able to chelate other nutrients, while in ruminants it is lysed by bacterial flora), leading to improvements in the nutritional value and processing properties of okara. Fungi, bacteria and yeasts are therefore very important for the production of functional ingredients and food products (Figure 6) [150].

### 5.1. Fungal Fermentation

SB pulp is suitable for fungal fermentation as it provides a physical surface for the adhesion and growth of fungi. Filamentous fungi excrete cellulolytic enzymes, including endoglucanase, esoglucanase and β-glucosidase, breaking down lignocellulosic biomass and thus promoting better digestibility.

#### 5.1.1. Production of Bioactive Compounds

The fungal fermentation of okara has been studied for the extraction of bioactive substances. Fujita, Funako and Hayashi have fermented okara with *Aspergillus* sp. HK-388, a strain isolated from soil samples in Osaka (Japan) [152]. The bioactive compound that was isolated in the methanolic extracts was 8-hydroxydaidzein. 8-Hydroxydaidzein can inhibit aldose reductase and tyrosinase, indicating that it has potential pharmaceutical and cosmetic applications. Since it was not detected in unfermented okara, the authors assumed that it was a product of the biotransformation of daidzin and daidzein, both of which are present in the starting material. In another study [153], okara was fermented with *Monascus purpureus* IFRPD 4046, a red purple mould, with the aim of producing monacolin K, a hypolipidemic agent that has been approved in Europe [154,155]. The yield of monacolin K was 192 mg/kg okara, which is about 2.5 times lower than that obtained using rice as a substrate. A carbohydrate supplement may therefore be needed to improve the production of monacolin K from SCR.

Okara was also used as a substrate for the cultivation of edible mushrooms [156,157,158]. In these studies, a selected fungus was grown on okara and the fungal polysaccharide was then extracted from the fermented biomass. The results were then compared with those of the unfermented SCR extracts. Fermented okara extracts showed increased antioxidant capacity in vitro and, where in-vivo tests were performed, improved immunomodulatory activity [159,160]. It has also been shown that ultrasonic-assisted extraction improves the yield of fungal polysaccharides compared to hot water extraction. Li et al. (2016) suggested that fungal growth may degrade okara fibres to produce low molecular weight oligosaccharides and, at the same time, release antioxidant peptides from proteins [156]. This may lead to a synergistic effect between fungal polysaccharides and bioactive okara components in the extract.

#### 5.1.2. Production of Food Fermented by Fungi

SCR may be fermented by fungi to produce food for direct consumption. Meitauza, which is produced mainly in the province of Hubei, China, is an indigenous food that is based on fermented okara. Conventionally, steamed okara is shaped into blocks, cooled and covered with rice straws at a temperature below 20 °C for about 8–14 days to obtain natural fermentation [89]. *Actinomucor elegans* and *Zymomonas mobilis* are two microorganisms that are isolated from meitauza. In a study by Xu, Liu et al., pure crops of these microbes were inoculated to produce meitauza [161]. The final product showed a reduction in moisture content and crude protein, the degradation of okara dietary fibre and a significant increase in free amino acids. These compositional changes have led to a pleasant and delicate taste, as well as a smooth and rubbery texture.

Okara can also be used to prepare tempeh, a traditional Indonesian food, which is normally produced from whole SBs fermented by *Rhizopus oligosporus*. Okara-tempeh has been used as a dog snack in a study by Yogo et al. [162]. Significant increases in the concentrations of short-chain fatty acids and the levels of *Bifidobacterium* and *Bacillus* were observed after 1 week. The authors attributed this effect to SB oligosaccharides, such as raffinose and stachyose, which have prebiotic potential and can stimulate the growth of these bacterial species.

In Asia, SBs are typically fermented with *Aspergillus oryzae* as the first step in the preparation of several SB-based seasonings. SB biomass that is fermented with mould is known as koji. By replacing SBs with okara, okara-koji has been prepared, which, once dried, was added to biscuits and cupcakes [163]. The consistency and palatability of the resulting baked products, with up to 10% substitution in biscuits and 5% in cakes, were not affected. This pastry also showed reduced lipid oxidation and starch retrogradation during storage. Dried okara-koji was then used as a flour substitute to extend the shelf life of high-fat bakery products.

The similarity between the texture of okara and fermented SB paste (i.e., miso) led to a series of studies, by Matsuo and Takeuchi, on the health benefits and palatability of an okara-miso-based condiment [164,165]. The okara-miso was prepared in several steps: okara-oncom and soy-oncom were prepared for inoculation with intermediate *Neurospora*. A mixture of 10% okara-oncom and 90% soy-oncom was then fermented with a commercial starter, *A. oryzae* [166]. Compared to miso produced entirely from steamed SBs, okara-miso showed greater antioxidant activity and greater anti-mutagenicity. In-vivo studies have also shown that rats fed with okara-miso had significantly higher hepatic catalase activity, significantly lower serum cholesterol and lower liver thiobarbituric acid reactive substance values than rats fed with normal miso [167]. It has been suggested that the antioxidant effect is due to the higher amounts of isoflavonic aglycones present in the okara-miso. The digestible aglycones and proteins are therefore able to facilitate the decomposition and removal of cholesterol, with the consequent effect of lowering it. The organoleptic properties of foods prepared with okara-miso have been defined as “acceptable”. This is probably due to the addition of higher quantities of aspartate and glutamate to give a characteristic umami flavour and possibly mask the bitterness of the aglycones.

**Figure 7:**
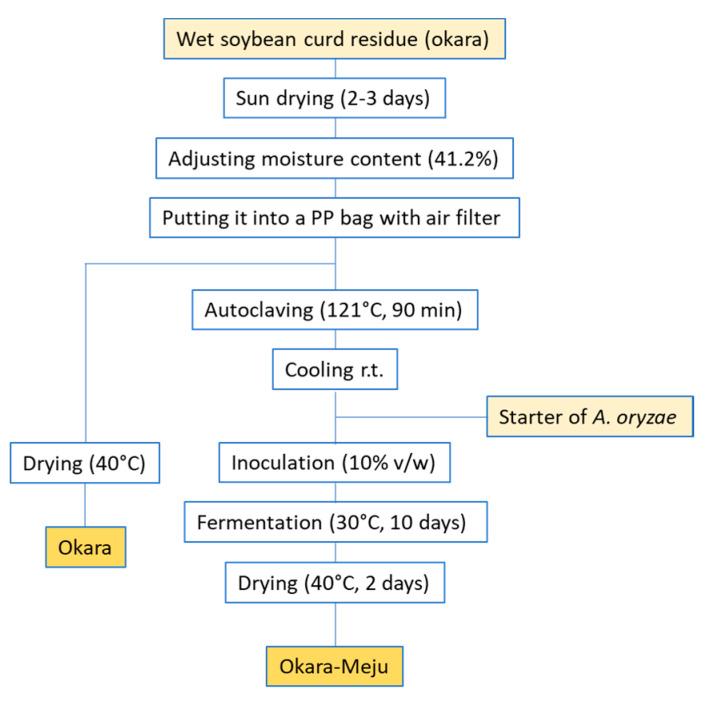
Procedure for the preparation of fermented SCR (okara-meju).

Meju is a traditional Korean SB cake that has been fermented and dried naturally, using *A. oryzae* [168]. Lee et al., following the principles of meju production, replaced SBs with okara (Ok) to obtain okara-meju (Ok-Me) [169]. Its dietary effects on body weight, blood lipids and antioxidant activity in mice that were fed on a high-fat diet (HC) were then examined. After an 8-week treatment, the body-weight gains of the Ok and Ok-Me groups were significantly lower than that of HC one, with the Ok-Me group value being lower than that of the Ok group. Furthermore, the serum TG, total-cholesterol and LDL-cholesterol contents of Ok and Ok-Me groups were lower than those of HC (Figure 7).

These studies highlight the additional health benefits that fermentation can confer to okara, helping to prevent obesity and improve lipid profiles.

### 5.2. Bacterial Fermentation

Most studies on the bacterial fermentation of okara involve the *Bacillus* species, probably because of its ability to produce extracellular alkaline proteases and the fact that it is commonly found in many fermented soy products [170].

#### 5.2.1. Production of Bioactive Compounds

Okara that is fermented with *Bacillus subtilis* has been shown to possess increased antioxidant activity in vitro. Proteinases produced by *B. subtilis* are able to hydrolyse soy proteins. It has also been proposed that the bioactivity of *Bacillus*-fermented okara may involve contributions from γ-polyglutamic acid [171], bioactive peptides [172] and the fibrinolytic enzyme nattokinase [173,174]. The optimisation of the fermentation and extraction of these bioactive components from fermented okara can provide greater insight into new potential therapeutic applications.

The inhibition of α-glucosidase from fermented okara is another interesting area of research. α-Glucosidase catalyses the breakdown of starch and disaccharides into glucose, meaning that its inhibition can reduce the absorption of food carbohydrates. α-Glucosidase inhibitors are one of the crucial therapeutic agents for hyperglycaemia-related diseases, such as diabetes and obesity. Zhu et al. have examined the inhibition of α-glucosidase by some microorganisms that are used to produce fermented okara [175]. *B. subtilis* B2 showed more than 90% inhibitory activity at a concentration of 0.625 mg/mL (methanol extract) and 0.313 mg/mL (aqueous extract), as opposed to the very low inhibitory activity observed in unfermented okara extracts. Subsequently, Zhu et al. purified and identified 1-deoxyrimycin (DNJ) as the α-glucosidase inhibitor in a fermented okara suspension in 2010 [176]. 1-DNJ, a naturally occurring nitro-derivative of sugar, and its derivatives are potential therapeutic agents in the management of diabetes, HIV infection and Gaucher’s disease [177]. Based on the maximum inhibitory activity against α-glucosidase, the optimal yield of 1-DNJ in an aqueous mixture containing fresh okara at 4.5% (*w*/*v*) was 0.74 mg/g fermentation broth (dry basis). The industrial synthesis of 1-DNJ is generally based on a combined chemical-biotechnological approach [178], and the possible use of okara as an economical raw material for the production of 1-DNJ for food use is extremely interesting.

9-*Cis*-11-*trans* conjugated linoleic acid is another noteworthy bioactive compound that is obtained from the fermentation of SCR. In 2010, Vahvaselka and Laakso were the first to exploit the linoleic acid fraction of okara by hydrolysing it with lipolytic oatmeal for 3 weeks, then fermenting it with *Propionibacterium freudenreichii ssp. Shermanii* in an aqueous okara suspension at 5% *w*/*w* [179].

#### 5.2.2. Using Dried Okara as A Prebiotic

When consumed, prebiotics selectively stimulate the growth and/or activity of certain intestinal microbes that can confer health benefits to the host [180]. The use of okara as a prebiotic has been investigated in in-vitro studies using *Bifidobacterium bifidum* and *Lactobacillus acidophilus* [93,181]. Okara provided a surface for bacteria-cell adhesion, thus facilitating substrate absorption and cell growth. Treatment with β-glucanase (Ultraflo L^®^) increased the content of okara-soluble dietary fibres and subsequently increased fermentation by *B. bifidum* [182]. The conversion of the insoluble dietary fibres of okara into soluble fibres was also observed when *Streptococcus thermophilus* and *Lactobacillus delbrueckii* subspecies *bulgaricus* were used [183].

Several researchers have studied the dietary effects of SB yoghurt, produced via the lactic fermentation of soymilk and okara, on lipid and cholesterol blood levels in rats [184]. This yoghurt was prepared by mixing soymilk and dried okara powder, in a 2:1 ratio, and then fermenting the mixture with *L. delbrueckii* subspecies *delbrueckii*. The final product was then freeze-dried and incorporated into rat diets. Regardless of their diet, rats fed with this SB yoghurt had a significantly lower level of total plasmatic cholesterol than the control group and groups fed only with a standard soymilk yoghurt or with a mixture of unfermented soymilk and okara. This suggests that the use of okara provided additional benefits to soymilk, as the fibre-rich okara facilitated the excretion of bile acids, through the adsorption on faecal matter, thereby improving the hypocholesterolaemic effect [184,185]. Moreover, fermentation played a key role in the cholesterol-lowering effect as it caused the production of bioactive peptides via the enzymatic hydrolysis of soy proteins. Results of DNA micro-array analyses also showed that the consumption of this SB yoghurt reduced lipid and cholesterol synthesis and stimulated the β-oxidation of fatty acids and cholesterol disintegration.

The organoleptic qualities and structural profiles of SB yoghurt were then evaluated. Soymilk with either dried okara alone or inulin (a carbohydrate polymer indigestible to humans, classified as soluble fibre) and dried okara were fermented with a starter culture of yogurt that contained *L. acidophilus*, *Bifidobacterium animalis* subsp. *lactis* and *S. thermophilus* [116]. These yoghurts showed significantly higher physical stability, but a low pleasantness index, probably due to the relatively large size of the dried okara particles. Nevertheless, the addition of inulin seems to increase palatability.

### 5.3. Fermentation Using Yeasts

The strong metabolic activity and diversity of yeasts mean that they offer great potential for the biotransformation of SCR. In fact, the use of okara fermentation by yeasts has so far been concentrated mainly on final consumer products, with the aim of providing better nutritional or aromatic effects.

In 2011, Rashad et al. studied the feasibility of producing a yeast-fermented okara food and paid particular attention to improving nutritional quality [186]. A mixture of yeasts, *Candida albicans*, *Candida guilliermondii*, *Kluyveromyces marxianus* NRRL Y-7571 and NRRL Y-8281, *Pichia pinus* and *Saccharomyces cerevisiae,* was inoculated in the okara to evaluate solid state fermentation (SSF). Yeast fermentation generally increased the amount of protein and ash and reduced the content of fibre, carbohydrates and crude lipids. The changes were caused by the metabolic activities and action of extracellular yeast enzymes. The in-vitro antioxidant activities of the fermented product were also about 1.5–2 times higher than those of the control.

**Figure 8:**
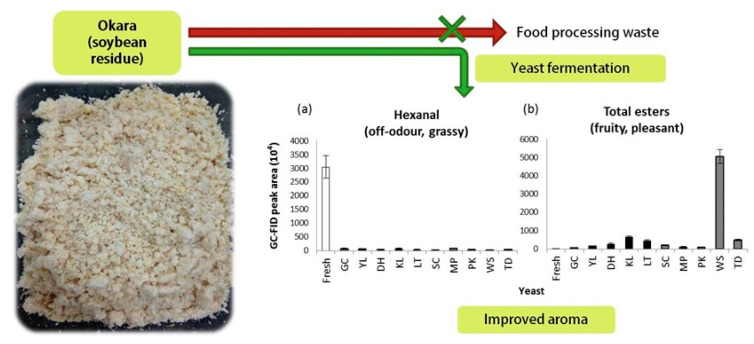
Changes in (**a**) hexanal and (**b**) total esters in fresh and yeast-fermented okara (GC = *G. candidum*, YL = *Y. lipolytica*, DH = *D. hansenii*, KL = *K. lactis*, LT = *L. thermotolerans*, SC = *S. cerevisiae*, MP = *M. pulcherrima*, PK = *P. kluyveri*, WS = *W. saturnus*, TD = *T. delbrueckii*) (Image credit: VONG Weng Chan) [188].

Okara was also individually fermented with ten different yeasts in an SSF, and changes in the aroma profile were then evaluated [187]. The yeasts used were typically associated with dairy products (*Geotrichum candidum*, *Yarrowia lipolytica*, *Debaryomyces hansenii*, *Kluyveromyces lactis*) and wines (*S. cerevisiae*, *Lanchancea thermotolerans*, *Metschnikowia pulcherrima*, *Pichia kluyveri*, *Torulaspora delbreuckii*, *Williopsis saturnus*), collectively referred to as “dairy yeast” and “wine yeast”, respectively. This study exploited the proteolytic and lipolytic qualities of dairy yeast and the ability of wine yeast to produce esters. After fermentation, the main odorous molecules present in fresh okara, saturated and unsaturated C6 aldehydes, were transformed into methyl ketones and/or esters, and the unwanted, grassy okara odour was greatly reduced. In particular, the okara fermented by *W. saturnus* contained the largest quantity and widest variety of esters, particularly C6 esters, and a significantly fruity odour (Figure 8). This study illustrates the possibility of obtaining esters and modifying the aroma of okara, via biotransformation, to improve its organoleptic quality.

The direct fermentation of okara, sometimes with the addition of nutrients, however, is not the only way forward. There have also been numerous studies on the compositional modification of raw okara, including physical processing methods (e.g., high hydrostatic pressure, extrusion), as well as chemical and enzymatic treatments [14,189,190,191]. The general increase in the amount of soluble dietary okara fibres following these treatments highlights their feasibility as a pre-fermentation step, increasing the amount of fermentable carbohydrates and oligosaccharides.

Studies have also been conducted on the fractionation of specific components of okara, such as dietary fibres, proteins and isoflavones [96,192,193,194,195,196]. This contributes both to the biovalorisation of these isolated components and to higher efficiency and yield than the direct fermentation of okara.

Finally, microbial transformation of the protein fraction, or its hydrolysates, can produce active peptides and single-cell proteins, while fermentation of the lipid fraction, using lipolytic and oily microorganisms, can be exploited for the production of microbial oils and other substances that are derived from lipids.

## 6. Nutrition and Health

As mentioned above, okara contains high levels of dietary fibre and proteins, as well as significant amounts of isoflavones and mineral elements, which gives it a high nutritional value and a potential prebiotic effect. It is therefore potentially useful as a functional ingredient with health-promoting properties [195].

### 6.1. Diabetes

In recent decades, the incidence of diabetes, particularly type 2, which accounts for more than 90% of cases, has increased rapidly (from 151 million in 2000 to 285 million in 2010). By 2030, the overall prevalence of diabetes will be 7.8%, with about 438 million adults suffering from it [197]. Researchers believe that consuming large amounts of soluble fibre can help control blood sugar levels after meals as it slows down the rate of carbohydrate uptake in the intestine. Soluble fibre also reduces blood cholesterol and triglyceride (TG) levels by binding to cholesterol and helping to eliminate it from the body. In addition, a protein-rich diet may be useful for people with hyperglycaemia [198]. Okara, which contains about 50% fibre and 25% protein, is an ideal food for diabetics. In fact, it has been used for many years by the Chinese population to prevent this chronic disease.

In 2000, Xu et al. studied the effect of SB fibres on the blood sugar, lipid levels and hepatic-nephritic histomorphology of mice with streptozotocin-induced diabetes [199]. The glycaemia, total serum cholesterol and TGs of diabetic mice fed with okara-containing food for 5 weeks decreased significantly, while high-density lipoprotein (HDL) cholesterol increased significantly. Their study showed that okara can significantly reduce the plasma levels of sugar and lipids, improve blood sugar and lipid metabolism, and protect the liver and kidneys of diabetic mice.

### 6.2. Hyperlipidaemia

Okara may play an interesting role in preventing hyperlipidaemia. In a study by Villanueva et al. [34], male Syrian golden hamsters were fed a high-fat diet supplemented with okara for 3 weeks. The different diets contained either 13% or 20% okara fibres (Ok-13 and Ok-20), low protein okara with 13% fibre (Ok1-13) and isoflavone-free okara with 13% fibre (Ok2-13). The plasma levels of TGs, VLDL plus LDL cholesterol, and total cholesterol in hamsters fed with OK-20 decreased significantly. Total lipid, TG, total and esterified cholesterol concentrations in the liver were reduced by the OK-20 diet. All tested okara diets increased the faecal excretion of total lipids, TGs, free cholesterol and total nitrogen. The results suggest that the main components of okara, dietary fibre and protein, may be related to total-lipid and cholesterol reduction in plasma and the liver, as well as increased faecal production in hamsters fed a high-fat diet.

Wang and Li studied the effect of okara fibre on lipid metabolism and the hemorheology of rats fed with a cholesterol-rich diet [200]. In groups fed with either okara or pectin, total cholesterol and LDL cholesterol levels decreased significantly after 8 weeks, while the pure cellulose-fed group had no hypolipidemic effect. In all groups, TGs and HDL cholesterol underwent no noticeable changes. Therefore, a diet containing either 6% okara or pectin may reduce blood viscosity and platelet aggregation in hyperlipidaemic rats. More specifically, SCR showed a more pronounced effect on these aspects than pectin.

### 6.3. Obesity

Okara may be effective as a weight-loss dietary supplement with a potential prebiotic effect. Préstamo et al. have reported that growth rate and nutritional efficiency were lower in an okara-fed group than in a control group, even though SCR, in the form of a dietary supplement, had no influence on the food intake in rats [201]. An increase in faecal weight and moisture was observed in the SCR-fed group. Moreover, the in-vivo colonic fermentation of okara resulted in a lower pH and the higher total production of short-chain fatty acids, demonstrating that it might be effective as a weight-loss dietary supplement with a potential prebiotic effect.

In another study, mice were fed either a high-fat diet (14% crude fat) or a high-fat diet supplemented with dry okara (10%, 20% or 40%) for 10 weeks [202]. The intake dose of okara suppressed the development of body weight and white epididymal adipose tissue in a dependent manner, and prevented an increase in plasma lipids, including total cholesterol, LDL cholesterol and unesterified fatty acids. Consuming okara also prevented liver steatosis. The real-time reverse-polymerase transcriptase chain reaction (RT-PCR) revealed that the intake of okara led to an under-regulation of the fatty-acid synthesis gene in the liver and an overregulation of the 7α-hydroxylase cholesterol gene.

### 6.4. Antioxidant Activity

In-vitro experiments have shown that okara is a potential source of antioxidant components, [98], in particular protease hydrolysate [97]. Zhu et al. have studied the antioxidant activity of meitauza, a traditional Chinese food derived from SB residue that is fermented by *Bacillus subtilis* B2 [175]. Compared to the aqueous soy koji extract, the aqueous okara-koji extract showed higher scavenging activity against the 1,1-diphenyl-2-picrylhydrazyl radical (DPPH).

The alkali-soluble polysaccharide fractions extracted from okara showed in-vitro antioxidant power (11–26 μmol Trolox equivalent (TE)/g dry weight) and free-radical scavenging activity (63–78 μmol TE/g dry weight). The potential antioxidant activity of the polysaccharides in the okara cell wall can be attributed to pectins, although the contribution of residual proteins cannot be excluded [26].

An ethanolic okara extract has shown greater scavenging activity against DPPH radicals than petroleum ether, ethyl acetate and acetone extracts. Synergism with vitamin C and citric acid was also reported [203].

## 7. Limiting Factors for Okara Valorisation

Although the worldwide production of okara is high, its utilisation index is low, which means poor yields from rich resources. More worryingly, management mistakes are causing significant environmental pollution. Some of the factors that limit its efficient use are summarised below.

### 7.1. Humidity and Drying

Despite the wealth of nutritional components, the high moisture content of okara, between 70% and 80%, and the 25% protein (dry base) hamper proper preservation. As a result, it decomposes rapidly once produced [204]. To overcome these limitations, the fresh residue should be dried as soon as possible, under appropriate drying conditions, to facilitate its movement and transport. The natural drying process is not suitable because of the time required and the influence of the weather, meaning that the product begins to rot before drying is complete. General preservation methods for okara include freezing (low temperatures from 0 °C to −18 °C or lower inhibit the microorganisms’ growth), oven drying and vacuum freeze-drying.

Indirectly heated conductive drying under agitation is a good choice for drying the residue, which is initially in the form of sticky lumps but disintegrates into small pieces or particles during drying [205]. Cui and Luo used a flash dryer with a rotating mill to dry okara and obtained satisfactory results [206].

Taruna and Jindal have studied the use of a continuous fluidised bed of inert particles, similar to a vortex, to dry SB pulp [204]. Although this drying technique provided acceptable results, both in terms of drying kinetics and energy consumption, the process suffered from okara agglomeration when a high okara-feeding speed was used; the residue, in fact, joined the inert particles together to form lumps, resulting in reduced drying rates.

According to a study by Wachiraphansakul et al. SCR that is dried in a fluidised bed has acceptable qualities in terms of colour, protein content and, above all, urease activity, when a water jet dryer is used [207]. However, a limitation in the blower used means that only a small amount of okara can be introduced into the drying chamber, leading to unsatisfactory specific energy consumption values. The addition of silica gels, used as absorbent particles, has been found to facilitate the drying process, both in terms of drying kinetics and the quality of the dried okara [84].

Treatment with a high voltage electric field can significantly improve drying speed, compared to kiln drying [208]. In fact, the drying time under high electric field conditions has been reduced by 15–40%, compared to the control [209]. Microwave vacuum drying is a potential and competitive option for drying okara because of its fast drying and low volume. This combined drying method has a drying time that is 90% lower than that of hot-air drying and freeze drying, while the quality of the dried product is similar to that obtained by freeze drying [210].

Drying methods have a significant effect on the sensory qualities and functional properties of okara. In terms of water-retention capacity, swelling capacity and the ability to bind lipids, freeze-dried SCR has proven to be the best, followed by vacuum drying and hot air drying. On the other hand, in terms of cation exchange capacity, hot-air-dried okara is the best, followed by vacuum drying and freeze drying [211]. However, all the common drying methods are energy intensive and cause major changes to the taste, colour and aroma of the product.

### 7.2. Anti-Nutrients

Raw SBs have high anti-nutritional factors, in particular, trypsin inhibitors that are partially inactivated during the solvent extraction and the roasting process [212,213]. Trypsin inhibitors are one of the most important factors that limit the application of okara in feed. Cattle fed with okara have digestion problems due to the antitrypsin, a further factor that limits its use in animal development. Experimental results show that fresh okara, used as a direct feed, has an impact on growth, behaviour and physiological activity [214]. Inactivation methods for trypsin inhibitors include physical, chemical, bioreduction and fermentation processes, as well as complex methods that make use of natural compounds [215].

## 8. Conclusions

This review examines the composition of dry SCR, its health functions, uses, limiting factors and current warnings, with the aim of stimulating the valorisation of these by-products.

The disposal of okara is still an unsolved problem caused by the increased global production of soymilk, tofu and their derivatives. Although numerous studies have been carried out on this topic, only a few propose a means of cost-effectively exploiting SCR. Furthermore, most tofu and soymilk factories are small and widely dispersed, making it difficult to collect an abundant quantity of okara for rapid, centralised processing before it degrades. The development of suitable dryers is therefore the fundamental prerequisite for this substance’s full exploitation.

Although SCR can be used in many contexts, it is also important to adopt appropriate methods for its use. Drying and grinding into a powder, then mixing with wheat flour or other ingredients to prepare composite flours, for the nutrition of people with specific needs (such as obesity and diabetes), or functional foods is an interesting choice for its use. Moreover, chemical and enzymatic treatment, fermentation, extrusion, high pressure and micronisation can improve the nutritional quality, taste and processing properties of okara and consequently the acceptance of the food as a whole. Future long-term randomized clinical trials are needed to confirm the healthy benefits of nutraceutical components and functional foods from SCR. In addition, pharmacodynamic studies will be useful to understand the mechanisms of action of polyphenols, isoflavones or bioactivated peptides isolated from SCR. In this regard, more efficient extraction processes and standardized protocols will guarantee high-quality products for food and nutraceutical industrial preparations.

In conclusion, although considerable progress has been made in recent years, the development of devices for the proper conservation of SCR and techniques for its proper use are challenges for the future. According to “circular economy” principles, transforming by-products from waste materials into in new added-value products is an ever more urgent task.

## Figures and Tables

**Table 1:** Nutritional composition of traditional unfermented soy-based foods, such as fresh, dried and boiled soybeans (SBs), soy flour, SBs meal, soy protein (SP) concentrate and isolate, soymilk and okara (100 g portion, based on USDA Nutrient Database) [12].

Compound	SBs	SBs	SBs	Soy	SB	SP	SP	Soy	Okara ^9^
(Amount Unit/100 g)	Raw ^1^	Dried ^2^	Boiled ^3^	Flour ^4^	Meal ^5^	conc. ^6^	Isolate ^7^	Milk ^8^	
Water (g)	8.54	0.8	62.6	5.2	6.94	5.8	4.98	93.3	81.6
Energy (kcal/KJ)	446/1866	449/1880	172/721	434/1816	337/1409	328/1373	335/1401	33/138	76/320
Protein (g)	36.5	43.3	18.2	37.8	49.2	63.6	88.3	2.8	3.52
Total lipid (g)	19.9	21.6	8.97	206	2.39	0.46	3.39	2.0	1.73
Tot saturated FA (g)	2.88	3.13	1.30	2.99	0.27	0.05	0.42	0.21	0.19
Tot monounsaturated FA (g)	4.40	4.78	1.98	4.56	0.41	0.08	0.64	0.33	0.30
Tot polyunsaturated FA (g)	11.3	12.2	5.06	11.7	1.04	0.20	1.65	0.83	0.76
Ash (g)	4.87	5.28	1.91	4.46	5.58	4.7	3.58	0.27	0.88
Carbohydrate (by diff., g)	30.2	29.0	8.36	31.9	35.9	25.4	0	1.8	12.2
Fibre (total dietary, g)	9.3	8.1	6	9.6	NR	5.5	0	1.3	NR
Sugars (total, g)	7.33	NR	3	7.5	NR	20	0	NR	NR
*Minerals*									
Calcium (mg)	277	140	102	206	244	363	178	4.0	80
Iron (mg)	15.7	3.95	5.14	6.37	13.7	10.8	14.5	0.58	1.3
Magnesium (mg)	280	228	86	429	306	140	39	19.0	26
Phosphorus (mg)	704	649	245	494	701	839	776	49.0	60
Potassium (g)	1.80	1.36	0.52	2.5	2.49	0.450	0.081	0.141	0.213
Sodium (mg)	2	2	1	13	3	900	1005	12	9
Zinc (mg)	4.89	4.77	1.15	3.92	5.06	4.4	4.03	0.23	0.56
Copper (mg)	1.66	1.08	0.41	2.92	2	0.98	1.60	0.12	0.2
Manganese (mg)	2.52	2.18	0.82	2.28	3.8	4.19	1.49	0.17	0.40
Selenium (µg)	17.8	19.3	7.3	7.5	3.3	0.8	0.8	1.3	10.6
*Vitamins*									
Ascorbic acid (C) (mg)	6	4.6	1.7	0	0	0	0	0	0
Thiamine (B1) (mg)	0.87	0.43	0.16	0.58	0.69	0.32	0.18	0.161	0.02
Riboflavin (B2) (mg)	0.87	0.78	0.28	1.16	0.25	0.14	0.1	0.07	0.02
Niacin (B3) (mg)	1.62	1.06	0.40	4.32	2.59	0.72	1.44	0.15	0.1
Pantothenic acid (B5) (mg)	0.79	0.47	0.18	1.59	1.98	0.06	0.06	0.05	0.09
Pyridoxine (B6) (mg)	0.38	0.22	0.23	0.46	0.57	0.13	0.1	0.04	0.12
Folate (B9) (µg)	375	205	54	345	303	340	176	1.5	26
Retinol (A) (IU) *	22	0	9	120	40	0	0	10	0
α-Tocopherol (E) (mg)	0.85	NR	0.35	1.95	NR	NR	0	0.01	NR
Phylloquinone (K) (µg)	47	37	19.2	70	NR	0	NR	NR	NR

* 1 IU = 0.3 mcg retinol. NR = not reported, FA = fatty acids. ^1–9^ Please see ref. [12], full description, Food Data Central Identifier (FDC ID), National Nutrient Database Identifier (NDB ID) for the Standard Reference Legacy Release (SR Legacy) of all soy-based foods are cited in the corresponding reference [12].

**Table 2:** Nutritional composition of traditional fermented soy-based foods, such as tempeh, miso, soy sauces, natto and tofu (100 g portion, based on USDA Nutrient Database) [14].

Compound	Tempeh ^1^	Miso ^2^	Natto ^3^	Soy Sauce ^4^	Tofu ^5^
(Amount Unit/100 g)					
Water (g)	59.6	43.0	55.0	66	70.0
Energy (kcal/KJ)	192/803	198/828	211/883	60/251	116/484
Protein (g)	20.3	12.8	19.4	10.5	8.92
Total lipid (g)	10.8	6.0	11	0.1	8
Tot saturated FA (g)	2.54	1.02	1.59	0.011	1.16
Tot monounsaturated FA (g)	3.2	1.12	2.43	0.017	1.77
Tot polyunsaturated FA (g)	4.3	2.88	6.21	0.044	4.52
Ash (g)	1.62	12.8	1.9	17.8	8.7
Carbohydrate (by diff., g)	7.64	25.4	12.7	5.6	4.38
Fibre (total dietary, g)	NR	5.4	5.4	0.8	NR
Sugars (total, g)	NR	6.2	4.9	1.7	NR
*Minerals*					
Calcium (mg)	111	57	217	20	46
Iron (mg)	2.7	2.49	8.6	2.38	1.98
Magnesium (mg)	81	48	115	40	52
Phosphorus (mg)	266	159	174	130	73
Potassium (g)	412	210	729	212	75
Sodium (mg)	9	3728	7	5586	2873
Zinc (mg)	1.14	2.56	3.03	0.43	1.56
Copper (mg)	0.56	0.42	0.67	0.14	0.38
Manganese (mg)	1.3	0.86	1.53	0.50	1.17
Selenium (µg)	0	7	8.8	0.8	17.3
*Vitamins*					
Ascorbic acid (C) (mg)	0	0	13	0	0.2
Thiamine (B1) (mg)	0.08	0.098	0.16	0.06	0.16
Riboflavin (B2) (mg)	0.36	0.23	0.19	0.152	0.10
Niacin (B3) (mg)	2.64	0.091	0	3.95	0.38
Pantothenic acid (B5) (mg)	0.28	0.34	0.22	0.38	0.13
Pyridoxine (B6) (mg)	0.22	0.20	0.13	0.2	0.09
Folate (B9) (µg)	24	19	8	18	29
Cobalamin (B12) (µg)	0.08	0.08	0	0	0
Retinol (A) (IU) *	0	87	0	0	0
α-Tocopherol (E) (mg)	NR	0.01	0.01	0	0
Phylloquinone (K) (µg)	NR	29.3	23.1	0	0

* 1 IU = 0.3 mcg retinol. NR = not reported, FA = fatty acids. ^1–5^ Please see ref. [14], full description, FDC ID, NDB N. (SR Legacy) of all the soy-based foods are cited in the corresponding reference [14].

**Table 3:** Composition of total fibre in okara (low molecular weight carbohydrates g/100 g dry matter).

Carbohydrates	References		
	[25]	[26]	[27]
Rhamnose	0.85	0.3 ± 0.1	1.0 ± 0.1
Fuchose	0.45	0.5 ± 0.1	0.1
Arabinose	6.35	5.7 ± 0.1	-
Xylose	5.14	2.7 ± 0.1	-
Mannose	1.26	1.5 ± 0.3	-
Galactose	10.83	10.4 ± 0.2	0.2
Glucose	15.01	11.9 ± 0.4	0.2
Sucrose	-	-	0.6 ± 0.1

**Table 4:** Amino acid composition (mg/g of protein) of unmodified okara protein isolate.

Amino Acids	Content
Aspartic acid	117
Threonine	41
Serine	50
Glutamic acid	195
Glycine	46
Alanine	46
Cysteine + methionine	26
Valine	51
Isoleucine	51
Leucine	81
Tyrosine + phenylalanine	95
Lysine	65
Histidine	28
Arginine	75
Proline	36
Tryptophan	N.D.*

* Not determined.

**Table 5:** Contents of the twelve isoflavones in okara (mg per g dry weight).

Groups	Forms	Content (mg)
Aglycones	Daidzein	22
	Glycitein	1.1
	Genistein	31
β-glucosides	Daidzein	48
	Glycitein	2.2
	Genistein	53
Malonyl glucosides	Daidzein	64
	Glycitein	2.8
	Genistein	130
Acetyl glucosides	Daidzein	-
	Glycitein	3.2
	Genistein	-
Total		355

**Table 6:** Comparison of the chemical composition of okara and okara-based paste (100 g portion).

Nutrients	Okara	Formulations		
(Amount Unit/100 g)		F1	F2	F3
Humidity	80.25 ± 0.04	81.26 ± 0.04	81.42 ± 0.05	80.77 ± 0.06
Protein (g)	7.91 ± 0.25	3.07 ± 0.70	4.00 ± 0.26	4.72 ± 0.19
Lipids (g)	6.22 ± 0.45	5.62 ± 0.86	6.20 ± 0.09	7.62 ± 0.46
Ash (g)	0.86 ± 0.00	1.98 ± 0.00	1.76 ± 0.01	1.72 ± 0.00
Total fibre (g)	13.83 ± 0.49	5.79 ± 0.17	7.17 ± 0.22	8.00 ± 0.25
Soluble fibre (g)	3.25 ± 0.09	1.67 ± 0.03	1.99 ± 0.04	2.19 ± 0.05
Insoluble fibre (g)	10.58 ± 0.40	4.13c ± 0.14	5.18 ± 0.18	5.82 ± 0.20
Carbohydrates (g)	2.44 ± 1.29	5.8 ± 0.49	4.61 ± 0.32	3.50 ± 1.37
Energy (kcal)	100.17	89.65	94.20	105.81
